# Gender disparities in lost productivity resulting from non-communicable diseases in Mexico, 2005–2021

**DOI:** 10.7189/jogh.14.04121

**Published:** 2024-05-31

**Authors:** Carlos M Guerrero-López, Edson Serván-Mori, Stephen Jan, Laura Downey, Ileana Heredia-Pi, Emanuel Orozco-Núñez, María de la Cruz Muradás-Troitiño, Robyn Norton

**Affiliations:** 1 Center for Health Systems Research, The National Institute of Public Health, Cuernavaca, Morelos, Mexico; 2 The George Institute for Global Health, UNSW, Sydney, New South Wales, Australia; 3 The George Institute for Global Health, School of Public Health, Imperial College London, London, Scotland, UK; 4 Centre for Health Economics and Policy Innovation, Business School, Imperial College London, London; 5 Directorate of Sociodemographic Studies and Prospective. The National Population Council of Mexico, Mexico City, Mexico

## Abstract

**Background:**

Non-communicable diseases (NCDs) cause long-term impacts on health and can substantially affect people’s ability to work. Little is known about how such impacts vary by gender, particularly in low- and middle-income countries (LMICs), where productivity losses may affect economic development. This study assessed the long-term productivity loss caused by major NCDs among adult women and men (20–76 years) in Mexico because of premature death and hospitalisations, between 2005 and 2021.

**Methods:**

We conducted an economic valuation based on the Human Capital Approach. We obtained population-based data from the National Employment Survey from 2005 to 2021 to estimate the expected productivity according to age and gender using a two-part model. We utilised expected productivity based on wage rates to calculate the productivity loss, employing Mexican official mortality registries and hospital discharge microdata for the same period. To assess the variability in our estimations, we performed sensitivity analyses under two different scenarios.

**Results:**

Premature mortality by cancers, diabetes, chronic cardiovascular diseases (CVD), chronic respiratory diseases (CRD) and chronic kidney disease (CKD) caused a productivity loss of 102.6 billion international US dollars (Intl. USD) from 2.8 million premature deaths. Seventy-three percent of this productivity loss was observed among men. Cancers caused 38.3% of the productivity loss (mainly among women), diabetes 38.1, CVD 15.1, CRD 3.2, and CKD 5.3%. Regarding hospitalisations, the estimated productivity loss was 729.7 million Intl. USD from 54.2 million days of hospitalisation. Men faced 65.4 and women 34.6% of these costs. Cancers caused 41.3% of the productivity loss mainly by women, followed by diabetes (22.1%), CKD (20.4%), CVD (13.6%) and CRD (2.6%).

**Conclusions:**

Major NCDs impose substantial costs from lost productivity in Mexico and these tend to be higher amongst men, while for some diseases the economic burden is higher for women. This should be considered to inform policymakers to design effective gender-sensitive health and social protection interventions to tackle the burden of NCDs.

Although non-communicable diseases (NCDs) are the leading cause of premature death and disability worldwide, and most occurs in low and middle-income countries (LMICs), little is known about how gender impacts productivity loss in these countries [1].

Gender plays an important role in the ways in which individuals participate in the labour market, become ill, seek and access health care services, and suffer discrimination processes that lead to inequity. This social construction can exacerbate an unequal distribution of costs in terms of burden of disease, lost productivity, risk of impoverishment [2], and other related social problems because of gender-oriented labour division within the households and society. Rooted in stereotypes and societal expectations, gender defines the ways in which people assume their respective roles as men and women. This is relevant to public health because these socially accepted differences can negatively affect (health) outcomes, beyond issues of labour market participation and wage disparities [3]. Despite its prominent relevance, gender has not received full attention in the health literature and in the formulation of health policies [4].

Besides premature mortality and disability, NCDs impose considerable economic costs on individuals, households, health systems, and the entire social system. Based on the Cost of Illness Approach [5,6], studies have focused on analysing the direct costs associated with health care for NCDs (including doctor visits, medications, medical devices, physical therapies, and hospitalisation); nevertheless, the negative economic consequences go far beyond. Indirect costs encompass lost productivity caused by premature mortality, since individuals who die at a lower age than life expectancy stop contributing to society in terms of productivity.

Productivity is a measure of the efficiency of a person, business or country to convert inputs into useful outputs [7]. Productivity loss associated with diseases is also caused by absenteeism (when a person does not attend to their job because of demanding health care) and the time spent in seeking health care services, and presenteeism, which occurs when people are present at their labour place, but their productive capabilities are undermined because of illness. Furthermore, many health conditions require non-paid care by relatives and friends who incur considerable opportunity costs because the possibility of participating in the labour market is jeopardised. In most cases, women and girls make up greater than 75% of the unpaid care workforce [8,9] and thus sacrifice their personal and professional development. The relative importance of direct and indirect costs vary according to the nature of a disease in terms of duration, available technologies, disability caused and other factors [5].

In Mexico, the second most populous country in the Latin American and Caribbean region with almost 130 million people [10], the few studies about productivity loss caused by NCDs are usually related to other risk factors, such as tobacco [11], sugar-sweetened beverages [12] or air pollution [13,14]. However, to our knowledge, there is no comprehensive assessment of the lost productivity nor other social negative consequences caused by NCDs in Mexico with gender as a critical source of heterogeneity [15]. This heterogeneity stems from at least two factors: disparity in the burden of diseases between genders and the difference in the observed productivity between women and men. Both factors are related to socially constructed gender roles and stereotypes that can deepen gender inequity.

Based on the above-mentioned elements, our general objective was to estimate the economic cost of the lost productivity resulting from major NCDs among Mexican men and women in the context of a fragmented and segmented health system. Our specific objectives were to estimate the lost productivity caused by premature deaths and hospitalisation. We hypothesised that the economic burden of lost productivity was importantly determined by gender, a proven contributor to disparity.

## METHODS

We conducted an economic valuation of lost productivity based on the Human Capital Approach (HCA). We estimated lost productivity resulting from premature death and hospitalisations among adult women and men (20–76 years, which we assumed to be the life expectancy) in Mexico caused by the following ICD-10 codes: diabetes (E11-E14), cancers and neoplasms (chapters C and D), cardiovascular diseases - CVDs - (I05-I15, I60-I69), respiratory diseases - CRDs - (J40-J44), and chronic kidney disease - CKDs - (N18, N19). Under this approach, a crucial assumption is that earnings from labour correspond to marginal productivity in a competitive market [16]. The HCA is a widely accepted methodology for performing economic valuations of several health outcomes, such as premature mortality and acute disability due to illness [17].

### Productivity loss by premature mortality

The HCA relies on the notion that the flow of future annual productivity is lost because of premature mortality [18]. Our first primary source is the annual mortality microdata produced by the National Institute of Statistics and Geography of Mexico (INEGI for its acronym in Spanish) [19] during the period 2005 to 2021, which included more than 6.7 million death registries of adults aged 20–76 years. We constructed a pooled data set with official and exhaustive information for mortality.

The second main source was the National Employment and Occupation Survey (ENOE for its acronym in Spanish, collected quarterly by INEGI) [20]. ENOE is the main source of standardised information on the Mexican labour market, occupation, labour informality, underemployment, and unemployment, among population aged 15 y and over. It is a probabilistic, two-stage, stratified, clustered sample that allows for the generation of estimates at the national and state level within urban and rural strata. The sample size for the pooled data with all quarters from 2005 to 2021 (excluding the second quarter of 2020 due to lack of comparability caused by the COVID-19 lockdown) is 27 million observations. Of these, 51.7% correspond to women, and the rest to men. The analytic sample included 16.8 million observations for the participation stage and 6.6 million observations for the second stage, detailed below. These analyses were performed using the statistical software Stata v17MP, College Station, USA, 2021, considering complex design of the ENOE and sampling weights using the *svy* package module.

To estimate the productivity loss (*Yd*), we computed the present value of the sum of future expected productivities for every death according to age. We assumed that productivity equalled the income from labour (based on the ENOE), whether the work was formal or not. We used a two-stage model [21] to estimate the expected annual productivity (*Yage*) given age and gender. The first stage (or participation equation) was performed by estimating a probit model for participation (income from labour >0) that included age, gender, a dichotomic variable marital status, dichotomic variable of urban location (more than 2500 inhab.), State, year fixed effects and years of schooling as explanatory variables. We suppose that gender corresponds to sex, a sensible assumption since according to a recent survey, more than 99% of the adult population is self-reported as cisgender [22]. The second stage (the outcome equation for income from labour) consisted of a linear regression model adjusted by gender, age, squared age, years of schooling, State, year fixed effects and urban location, assuming 240 working days of eight hours per year. The expected productivity given the explanatory variables is defined as the product of the predicted probability of the first part by the predicted income from labour from the second part [21].

To calculate present values of future monetary figures, we discounted the expected productivities given age at a 3% annual rate [17] according to the following equation:



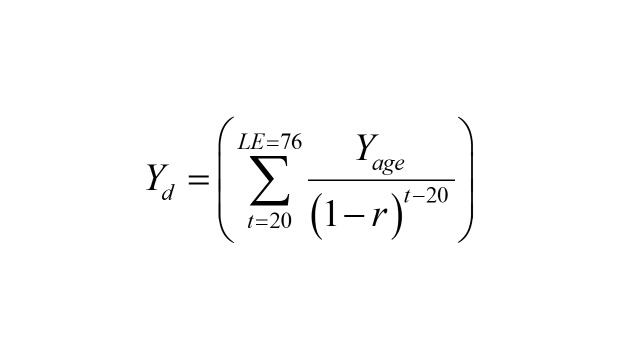



where: *LE* = life expectancy – we assumed 76 years, *Yage* = annual expected productivity at a certain age, *t* = age at death, *r* = discount rate. Present values of future productivity flows were then imputed according to the age, gender, and type of area (rural and urban) of individuals in the mortality microdata with valid values for these variables. To provide easier interpretation of the figures, we calculated the standardised rate of lost productivity by 100 000 inhab. using the projections of population by the National Population Council [10].

### Productivity loss by hospitalisations

Our two main sources of information are: 1) hospital discharge microdata published by the Ministry of Health [23], and 2) the ENOE. Hospital discharge microdata correspond to all hospitalisations occurred in public facilities, where the majority of hospitalisations take place in Mexico [24]. The number of hospitalisation events for all ages ascends to nearly 97.2 million from 2005 to 2021.

We calculated the lost productivity caused by morbidity using the same ICD-10 codes as in the case of premature mortality:



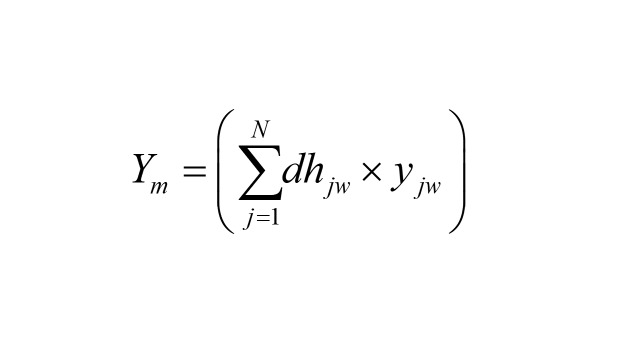



where *Ym* is the lost productivity caused by morbidity, *dh* are the days at the hospital for patients *j*, *w* is the disease of interest, and *y* is the expected daily income from work at every age among women and men estimated using the ENOE. It corresponds to the product of hourly income from work by eight (the legal length of a workday). The daily income from work at every age was estimated in the same fashion as in the case of premature mortality and inputted to the hospital discharge database according to age and gender.

To assess the variability in our estimations, we performed sensitivity analyses under two different scenarios. First, we assumed that both women and men faced the average expected productivity regardless the gender of individuals, and second, that the expected productivity of women was equal to men in order to account for opportunity costs for unpaid work [25]. By doing so, we assessed the productivity differences in the labour market because of the influence of gender.

All monetary units are expressed in International USD of 2021 using the purchasing power parity exchange rate of one international dollar (Intl. USD) = 10.4 Mexican Pesos (MXN) [26] and the Consumer Price Index [27]. A graphic overview of the estimation processes is provided in the **Online Supplementary Document**.

## RESULTS

### Productivity loss by premature mortality

**Figure 1:**
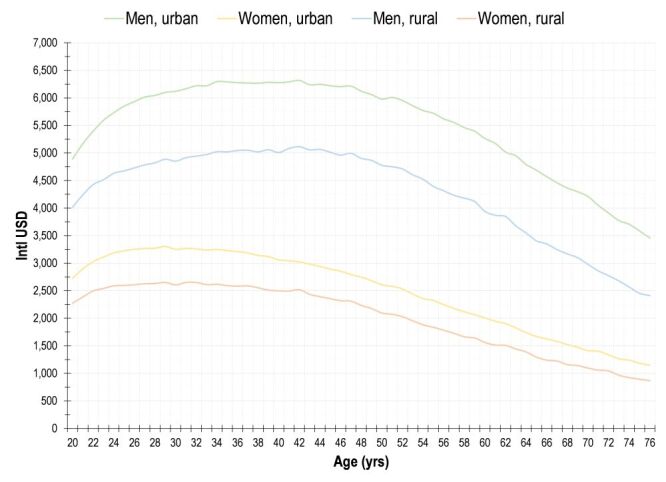
Mean expected annual income from labour by gender, location size, and age. Mexico, adults aged 20–76 years, 2005–2021.

We found that in the population aged 20–76 years, there is a significant difference in the predicted probability of labour participation between women and men: 31.6% for women whereas 57.7% for men. The expected annual income from labour according to gender and location size (the present value of forgone productivity and the expected hourly productivity by age, gender and location size estimated using the two-part model is available in the **Online Supplementary Document**) shows an inverted U-shape, as expected according to the human capital theory (**Figure 1**). Gender and location size are important variables that explain differences in income from labour.

**Table 1** shows that between 2005 and 2021, there were 2.8 million deaths caused by cancers and neoplasms, diabetes, CVDs, CRDs in CKDs in adult Mexicans (80.7% urban deaths, vs. 19.3% occurred in rural areas). Cancers and neoplasms caused the highest burden in terms of lost productivity since it accounted for 38.3% of the total. For cancers, the number of deaths is more significant among women and thus the lost productivity (535 923 vs. 467 020 deaths). Among women, cancers and neoplasms represent a higher proportion of lost productivity (47.0%). The lost productivity by premature mortality caused by diabetes was the second with 39.1 billion Intl. USD. This corresponds to 38.1% of the lost productivity caused by the set of NCDs considered. By gender, there were slightly more deaths among men (567 921 vs. 532 258). Chronic cardiovascular diseases are the third source of lost productivity (15.1%). The number of deaths and the amount of productivity loss is higher among men (228 344 vs. 204 932 and 11.8 billion Intl. USD vs. 3.7 billion Intl. USD, respectively). Chronic respiratory diseases represent 3.2% of the total productivity loss, and the burden in terms of the number of deaths and productivity loss is higher among men (78 546 vs. 55 421 and 2.64 vs. 0.7 billion Intl. USD, respectively). Finally, CKDs accounts for nearly 5.3% of the lost productivity, and for men the relative importance of this disease is higher than for women (5.7% vs. 4.3%). The total productivity loss caused by the set of NCDs considered in this study was calculated at 102.8 billion Intl. USD.

**Table 1:** Lost productivity caused by NCDs by premature mortality in Mexico, adults aged 20–76, by sex, 2005–2021

	Men						Women						Total
	**Urban**		**Rural**	**Total**	Scenario 1*	**Scenario 2†**	**Urban**	**Rural**	**Total**	Scenario 1*	**Scenario 2†**		
Cancers and neoplasms													
*Deaths*	377 446.0		89 574.0	467 020.0	467 020.0	467 020.0	440 165.0	95 758.0	535 923.0	535 923.0	535 923.0	1 002 943.0	
*Years of life lost*	6 202 096.0		1 415 521.0	7 617 617.0	7 617 617.0	7 617 617.0	8 051 655.0	1 766 661.0	9 818 316.0	9 818 316.0	9 818 316.0	17 435 933.0	
*Average years of life lost per death*	16.4		15.8	16.3	16.3	16.3	18.3	18.4	18.3	18.3	18.3	17.4	
*Lost productivity (million Intl. USD)*	22 282.6		3797.3	26 079.8	15 718.9	23 458.0	11 277.5	1953.0	13 230.5	20 346.5	30 389.3	39 310.4	
*% out of total*	34.9		35.8	35.0	35.4	35.1	47.7	43.4	47.0	46.4	46.0	38.3	
Diabetes													
*Deaths*	469 353.0		98 568.0	567 921.0	567 921.0	567 921.0	424 587.0	107 671.0	532 258.0	532 258.0	532 258.0	1 100 179.0	
*Years of life lost*	6 894 128.0		1 445 777.0	8 339 905.0	8 339 905.0	8 339 905.0	5 600 412.0	1 465 591.0	7 066 003.0	7 066 003.0	7 066 003.0	15 405 908.0	
*Average years of life lost per death*	14.7		14.7	14.7	14.7	14.7	13.2	13.6	13.3	13.3	13.3	14.0	
*Lost productivity (million Intl. USD)*	25 713.0		3986.0	29 699.1	17 497.3	26 503.8	7775.4	1590.7	9366.0	14 863.1	22 584.1	39 065.1	
*% out of total*	40.3		37.6	39.9	39.4	39.7	32.9	35.4	33.3	33.9	34.2	38.1	
Chronic cardiovascular diseases													
*Deaths*	181 904.0		46 440.0	228 344.0	228 344.0	228 344.0	161 785.0	43 147.0	204 932.0	204 932.0	204 932.0	433 276.0	
*Years of life lost*	2 776 075.0		616 440.0	3 392 515.0	3 392 515.0	3 392 515.0	2 202 095.0	540 055.0	2 742 150.0	2 742 150.0	2 742 150.0	6 134 665.0	
*Average years of life lost per death*	15.3		13.3	14.9	14.9	14.9	13.6	12.5	13.4	13.4	13.4	14.2	
*Lost productivity (million Intl. USD)*	10 117.4		1685.3	11 802.7	7063.7	10 596.6	3083.5	592.9	3676.4	5744.3	8651.0	15 479.1	
*% out of total*	15.8		15.9	15.9	15.9	15.9	13.0	13.2	13.1	13.1	13.1	15.1	
Chronic respiratory diseases													
*Deaths*	60 267.0		18 279.0	78 546.0	78 546.0	78 546.0	41 063.0	14 358.0	55 421.0	55 421.0	55421.0	133 967.0	
*Years of life lost*	568 846.0		158 118.0	726 964.0	726 964.0	726 964.0	370 100.0	130 515.0	500 615.0	500 615.0	500 615.0	1 227 579.0	
*Average years of life lost per death*	9.4		8.7	9.3	9.3	9.3	9.0	9.1	9.0	9.0	9.0	9.2	
*Lost productivity (million Intl. USD)*	2191.3		446.9	2638.2	1551.5	2367.4	518.9	142.2	661.1	1071.5	1637.4	3299.3	
*% out of total*	3.4		4.2	3.5	3.5	3.5	2.2	3.2	2.3	2.4	2.5	3.2	
Chronic kidney disease													
*Deaths*	49 519.0		13 204.0	62 723.0	62 723.0	62 723.0	36 751.0	10 126.0	46 877.0	46 877.0	46 877.0	109 600.0	
*Years of life lost*	1 020 465.0		261 548.0	1 282 013.0	1 282 013.0	1 282 013.0	711 146.0	196 024.0	907 170.0	907 170.0	907 170.0	2 189 183.0	
*Average years of life lost per death*	20.6		19.8	20.4	20.4	20.4	19.4	19.4	19.4	19.4	19.4	20.0	
*Lost productivity (million Intl. USD)*	3529.2		684.6	4213.8	2601.1	3828.3	995.5	217.1	1212.6	1852.1	2738.9	5426.4	
*% out of total*	5.5		6.5	5.7	5.9	5.7	4.2	4.8	4.3	4.2	4.1	5.3	
Total													
*Deaths*	1 138 489.0		266 065.0	1 404 554.0	1 404 554.0	1 404 554.0	1 104 351.0	271 060.0	1 375 411.0	1 375 411.0	1 375 411.0	2 779 965.0	
*Years of life lost*	17 461 610.0		3 897 404.0	21 359 014.0	21 35 014.0	21 359 014.0	16 935 408.0	4 098 846.0	21 034 254.0	21 034 254.0	21 034 254.0	42 393 268.0	
*Average years of life lost per death*	15.3		14.6	15.2	15.2	15.2	15.3	15.1	15.3	15.3	15.3	15.2	
*Lost productivity (million Intl. USD)*	63 833.5		10 600.1	74 433.7	44 432.5	66 754.0	23 650.7	4495.9	28 146.7	43 877.4	66 000.6	102 580.3	

NCD – non-communicable diseases, Intl USD – International United States dollars

*Total assuming average expected productivity.

†Total assuming average expected productivity for men.

**Figure 2:**
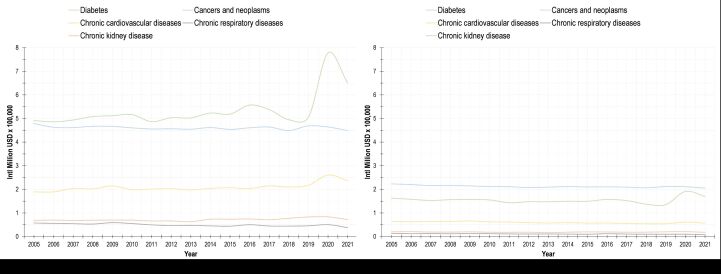
Trends in lost productivity by premature death caused by non-communicable diseases in Mexico, 2005–2021. **Panel A.** Men. **Panel B.** Women.

**Figure 2**, panels A–B shows the standardised rate of the lost productivity caused by premature death by gender and disease. The economic burden by the set of diseases included is higher among men. Among the latter, diabetes, CVDs, and CKDs have been increasing steadily. For both genders, cancers and neoplasms and diabetes represent the main source of lost productivity.

### Productivity loss by hospitalisations

The set of NCDs considered in this study caused 54.2 million days of hospitalisation, associated with 729.7 million Intl. USD of lost productivity: 477.3 for men and 252.4 for women. Cancers and neoplasms are the leading cause of lost productivity by hospitalisation (41.3%), followed by diabetes (22.1%), CKDs (20.4%), CVDs (13.6%) and CRDs (2.6%). Cancers and neoplasms have caused 8.6 million days for men and 14.3 million days for women, causing 167.4 and 134.2 million Intl. USD of productivity loss. Regarding diabetes, men have spent 6.2 million days at the hospital, while women 5.5 million days, accounting for 118.5 and 43.0 Intl. million USD of lost productivity respectively. Chronic cardiovascular diseases caused 3.8 million days at the hospital for men and 3.8 for women, causing 70.2 and 28.7 million Intl. USD of lost productivity. Among the diseases considered in the study, CRDs caused the least number of days at the hospital: 0.8 million days for men and 0.9 million days for women, and 13.0 and 5.8 million Intl. USD associated with productivity loss. Chronic kidney diseases caused 10.4 million days at the hospital, 5.5 for men and 4.8 for women, causing 108.2 and 40.7 million Intl. USD in productivity loss (**Table 2**).

**Table 2:** Lost productivity caused by NCDs by hospitalisation in Mexico, adults aged 20–76, by sex, 2005–2021

	Men		Women			Total
	**Observed productivity**	Scenario 1*	**Observed productivity**	Scenario 1*	**Scenario 2†**	
Cancers and neoplasms						
*Hospitalisations*	1 583 753.0	1 583 753.0	3 908 543.0	3 908 543.0	3 908 543.0	5 492 296.0
*Days*	8 613 087.0	8 613 087.0	14 304 310.0	14 304 310.0	14 304 310.0	22 917 397.0
*Average number of days*	5.4	5.4	3.7	3.7	3.7	4.2
*Lost productivity (million Intl. USD)*	167.4	116.6	134.2	208.4	296.3	301.6
*% out of total*	35.1	35.4	53.2	52.1	51.4	41.3
Diabetes						
*Hospitalisations*	1 024 056.0	1 024 056.0	1 020 902.0	1 020 902.0	1 020 902.0	2 044 958.0
*Days*	6 168 738.0	6 168 738.0	5 461 791.0	5 461 791.0	5 461 791.0	11 630 529.0
*Average number of days*	6.0	6.0	5.3	5.3	5.3	5.7
*Lost productivity (million Intl. USD)*	118.5	80.8	43.0	70.0	103.0	161.5
*% out of total*	24.8	24.6	17.0	17.5	17.9	22.1
Chronic cardiovascular diseases						
*Hospitalisations*	622 763.0	622 763.0	647 218.0	647 218.0	647 218.0	1 269 981.0
*Days*	3 813 342.0	3 813 342.0	3 772 170.0	3 772 170.0	3 772 170.0	7 585 512.0
*Average number of days*	6.1	6.1	5.8	5.8	5.8	6.0
*Lost productivity (million Intl. USD)*	70.2	47.8	28.7	46.8	69.0	98.9
*% out of total*	14.7	14.5	11.4	11.7	12.0	13.6
Chronic respiratory diseases						
*Hospitalisations*	145 156.0	145 156.0	166 242.0	166 242.0	166 242.0	311 398.0
*Days*	791 004.0	791 004.0	876 451.0	876 451.0	876 451.0	1 667 455.0
*Average number of days*	5.4	5.4	5.3	5.3	5.3	5.4
*Lost productivity (million Intl. USD)*	13.0	8.6	5.8	9.7	14.6	18.8
*% out of total*	2.7	2.6	2.3	2.4	2.5	2.6
Chronic kidney disease						
*Hospitalisations*	1 546 091.0	1 546 091.0	1 300 086.0	1 300 086.0	1 300 086.0	2 846 177.0
*Days*	5 542 675.0	5 542 675.0	4 832 332.0	4 832 332.0	4 832 332.0	10 375 007.0
*Average number of days*	3.6	3.6	3.7	3.7	3.7	3.6
*Lost productivity (million Intl. USD)*	108.2	75.1	40.7	64.8	93.7	148.9
*% among total*	22.7	22.8	16.1	16.2	16.3	20.4
Total						
*Hospitalisations*	4 921 819.0	4 921 819.0	7 042 991.0	7 042 991.0	7 042 991.0	11 964 810.0
*Days*	24 928 846.0	24 928 846.0	29 247 054.0	29 247 054.0	29 247 054.0	54 175 900.0
*Average number of days*	5.1	5.1	4.2	4.2	4.2	4.5
*Lost productivity (million Intl. USD)*	477.3	329.0	252.4	399.8	576.6	729.7

NCD – non-communicable diseases, Intl. USD – international United States dollars

*Assuming average expected productivity.

†Assuming men's observed productivity.

## DISCUSSION

This long-term economic validation revealed that NCDs caused 2.8 million premature deaths with an associated overall productivity loss of 102.6 billion Intl. USD from 2005 to 2021. This represents 4.1% of the Mexican Gross Domestic Product (GDP) in 2021 (2.5 trillion USD) [28]. Cancers and neoplasms (mainly among women) are the leading cause, followed by diabetes and CVDs (both more prominent among men). Regarding lost productivity caused by hospitalisations, the relative importance of the diseases is different: cancers and neoplasms are the first cause, particularly among women; diabetes is the second, CKD is the third, followed by CVD and CRD.

The productivity loss is higher among men than among women. The difference in lost productivity by mortality and morbidity by gender can be explained by disparities in the burden of disease (number of deaths and events, age of death or hospitalisation [29,30], number of days at hospital, etc.) and importantly, the differences in participation in the labour market and income from labour. To illustrate this point, regarding mortality, the number of deaths and years of life lost is higher among men. Consequently, our estimate of lost productivity was higher for men no matter if we assumed that the expected productivity of women was the same than men’s or using the overall average productivity by age both for women and for men. This holds for all the diseases included, except for cancers and neoplasms. For these, our estimate of lost productivity is higher for men (97% difference) despite the higher burden of disease for women, because of higher expected productivity for men. When we assumed equal expected productivity (whether average expected productivity or men’s productivity), the productivity loss becomes higher for women (22.8% higher). Similarly, we estimated that the productivity loss from hospitalisations is higher for men than for women (89% difference) despite the higher burden by hospitalisations and number of days at hospital for women. This difference is also due to the disparities in labour participation and wages. This, in turn, could be the consequence of gender stereotypes and even a reduced productivity for women-specific conditions such as menstrual symptoms [31], which requires further research in countries like Mexico. The disparity in expected productivity also points to that women suffer from child penalty in terms of reduced wages and lower probability of labour participation as reported elsewhere and as was suggested in **Figure 2** [32]. However, if we assumed equal productivity among genders (average expected productivity or men’s productivity), the lost productivity loss by hospitalisations would be higher for women (21.5% difference). Such disparity could be interpreted as a discrimination process and a result of gender roles and norms to the detriment of women [33].

The HCA assumes that individuals face a competitive labour market, with no discrimination, minimum transaction costs and no replacement of absent or lost workers. This implies that when a person dies, she/he will not be replaced and the economic output that the individual would have produced in the remaining life is lost. Despite the widespread use of this approach in economic evaluation studies, HCA has received several criticisms because its results could be overestimated when in fact the future economic output of a deceased person could be replaced by the work of a new person in the economy. The time until the production is replaced by another person is called the friction period. The frictional cost method to calculate productivity loss by premature mortality values only the lost output during the length of the friction period. Conversely, this length is specific for every economic activity and varies according to the level of employment or employment and the availability of employees with a similar level of skills and human capital. Because of these complexities and lack of information, the frictional cost method is employed at a lower extent [34]. Another criticism of the HCA is that it can have ethical implications because the economic valuations in different settings could result in radically different Figures [18]. For instance, the economic valuation of a premature death in a very low-income country could be a minimal fraction of a premature death at the same age that occurred in a high-income country [35]. Within the same country, there could also be different valuations depending on the area of the country and other variables such as gender [36], as we found. To this matter, we preferred to conduct the analysis in a positive way (in contrast to normative) and to discuss the difference of expected productivity by gender. To do so, we employed a two-stage regression model, which is preferred over a Heckman selection model when the purpose is to estimate the expected outcome at population levels and when using a large data set [37]. Despite these considerations and pitfalls, we argue that HCA remains as a main and widely accepted method to measure societal costs of illness and it is helpful to inform policymaking [34]. Furthermore, we relied on official data sources with census-nature and vast sample sizes for a long period, such as the ENOE or mortality and the hospital discharge microdata sets, which makes us confident that our results are robust. In addition, to our knowledge, this is the first study on the productivity loss caused by major NCDs in Mexico.

Our results showed a slight increase in productive loss at the end of the period analysed, specifically the years 2020 and 2021, which coincided with the beginning and the most intense phase of the COVID-19 in Mexico. This may be related to the well-established impact of this epidemiological contingency on NCDs. Numerous studies have documented excess mortality during this pandemic in Mexico, with the country suffering the seventh highest excess mortality rate in the world and the highest in the Latin American and Caribbean Region [38]. This was a result not only of deaths directly associated with COVID-19, but also of other causes related to NCDs, including ischemic heart disease, diabetes, and hypertensive diseases. This last result can be explained by the higher risk of developing severe COVID-19 and death from NCDs already present prior to the COVID-19 pandemic. The interruption and postponement of health care because of confinement measures, a reduction in the use of health services because of the fear of contagion in care facilities, and the resulting economic crisis [39,40].

Because of the lack of data availability, we did not consider costs other than lost productivity by mortality and morbidity. For instance, regarding hospitalisations, we did not include information on absenteeism after the hospital discharge, ie, the days that the individuals spent at home for recovery, nor the days spent by caregivers and we include hospitalisation only in the public sector, but these hospitalisations represent more than 80% of total hospitalisation in the country [24]. In addition, we did not include indirect costs by presenteeism nor productivity loss due to permanent disability caused by the set of diseases included. This also has an important gendered domain because men are potentially more likely to die from NCDs, whereas women tend to live longer in poorer quality of life [15]. Another caveat is that we only consider mortality and hospitalisations observed for the set of diseases included, without taking into account in our estimates the possible underrepresentation of women in the diagnosis and treatment of these diseases attributable to gender bias [41]. In this sense, our estimates should be interpreted as minimum bounds of the indirect costs of the diseases included.

An analysis by Rasmussen et al. (2016) provided global estimates of the economic impact caused by productivity loss from absenteeism, presenteeism, as well as early retirement attributable to 13 NCDs for the year 2015 [42]. The results varied widely but averaged approximately 6.5% of GDP, ranging from 5.4% of GDP in China to more than 8% (of GDP) in the US. In their study, the estimated productive loss for Mexico was 5.3% of GDP, less than that of other countries in the region such as Brazil (7.3%), Peru (7.0%), and Colombia (6.9%). The authors forecast an average increase of 0.6 percentage points in GDP by 2030, resulting from the convergence of several factors, including an aging workforce and the consequent high burden of chronic diseases, such as heart and respiratory diseases, strokes, and cancer. Our findings are very similar to those reported by Malkin et al. in 2022 [43], who estimated a productive loss of 4.5% for Saudi Arabia in a 2019 study. However, our results differed from theirs in several respects, specifically as regards the relative weight of each NCD analysed and scope of costs included. In Saudi Arabia, diabetes mellitus and major depressive disorder represented the NCDs primarily responsible for the productivity loss in that country, whereas in Mexico cancer and neoplasms were the main contributors to productive loss.

Regarding the time spent by caregivers, we provide an estimate and its related opportunity cost in the **Online Supplementary Document** from eight assistance activities for household members who need it, using data from the National Survey on Use of Time [44]. Ostensibly, most of the burden of these activities falls on women (75%), and the productivity loss in which women incurred ascended to more than 331 million Intl. USD in a year. The productivity loss is higher among women, regardless of the scenario of expected productivity used. These estimates cannot be directly linked to NCDs or specific conditions because the survey does not include those variables. However, it is plausible to assume that a considerable proportion comes from NCDs if we consider that, in largely traditional societies in Mexico and other in Latin American countries and the Caribbean, caregiving has come to be considered a gender role that falls on women [45]. This situation caused a serious decline in women’s participation in the labour market and a reduction of the available discretional time that women could devote to their personal and collective development and well-being [46]. Further research is needed to assess other undesirable economic consequences of NCDs that can deepen gender inequity because of socially constructed gender roles and stereotypes.

The establishment of a comprehensive care strategy in Mexico that allows for the construction of a National Care System should be considered a priority. Such a system would embody social responsibility through providing care for all people and guaranteeing services for the population in a context of dependency. These services must be sufficient, accessible, and of good quality, in addition to promoting people's autonomy and reducing gender inequalities [47]. The creation of a National Care System would offer tremendous benefits to women caregivers by opening numerous career opportunities in the educational, labour, social, and political spheres. It would also reduce the overload of unpaid work as a result of recognising women’s contributions, and work would be remunerated from within the care economy and entitle women to financial protection [47]. This becomes even more relevant if we consider that recent evidence suggests that the utilisation of outpatient services by Mexican adults requiring NCD care has been undermined by non-neutral gender inequalities for almost two decades, in part because of the segmented architecture of the Mexican public health system [48]. Consistent with these studies, we emphasise the need to consider these findings as a key factor in reorienting NCD health policies and programmes from a gender perspective.

This study offers two main lessons for LMICs and other countries with fragmented and segmented heath systems such as Mexico. First, in addition to direct health care costs, NCDs impose major societal costs, such as productivity loss that can threaten the economic development of families and the country. Second, we found that the burden of NCDs in terms of deaths, days of hospitalisation and productivity loss also varies greatly by gender and that this variability is caused mainly because of dissimilar burden of disease and importantly, from different expected productivity which is caused by discrimination processes and gender-based labour division. This is particularly salient in the case of cancers and neoplasms for women and diabetes and cardiovascular diseases for men. In the case of Mexico, it is also relevant to consider the influence of cultural factors that tend to normalise gender inequalities in the domestic, labour and social contexts [49].

## CONCLUSIONS

Our findings constitute overwhelming evidence for policymakers who should consider the enormous societal costs imposed by NCDs in a fragmented and segmented health system context, and design citizenship-based and gender-sensitive health and social protection programmes to tackle the burden of the NCDs, and the inequities caused by gender considered in this study [50]. In analysing productivity loss, it is essential to consider gender differences in the burden of disease and in the utilisation of health care services. Social contexts in the provision of care to the ill and the structure of the health system clearly create and exacerbate imbalances between men and women. These issues require a strong governance framework to assure that labour and social dimensions are being taken into account to enhance equity and to assure social opportunities for all in terms of labour participation, personal and professional development, financial protection and access to health care [50].

Gender-sensitive health and social protection interventions should take advantage of all opportunities to promote gender equality and reduce the gender-related inequities identified in studies of the economic impact of NCDs on productivity [51]. Policymakers should invest in capacity development to ensure that those who design and implement social protection interventions are sensitive to the role of gender, training public service providers to adopt culturally appropriate practices and increase awareness of the vulnerabilities caused by socially constructed stereotypes. Furthermore, it is crucial to ensure the active, equitable and effective participation of women, men, and non-binary genders in the administration of these gender-balanced interventions and programmes at all levels of (these) social protection programmes [47].

## Additional material:


Online Supplementary Document
