# Supplementary material for: Gender disparities in lost productivity resulting from non-communicable diseases in Mexico, 2005–2021
Source: J Glob Health. 2024 May 31;14:04121. doi: 10.7189/jogh.14.04121 (PMC11140423; doi:10.7189/jogh.14.04121)
Supplement: Online Supplementary Document [file jogh-14-04121-s001.pdf]

Online supplementary document

Appendix 1 - Figure A1. Overview of the estimation processes

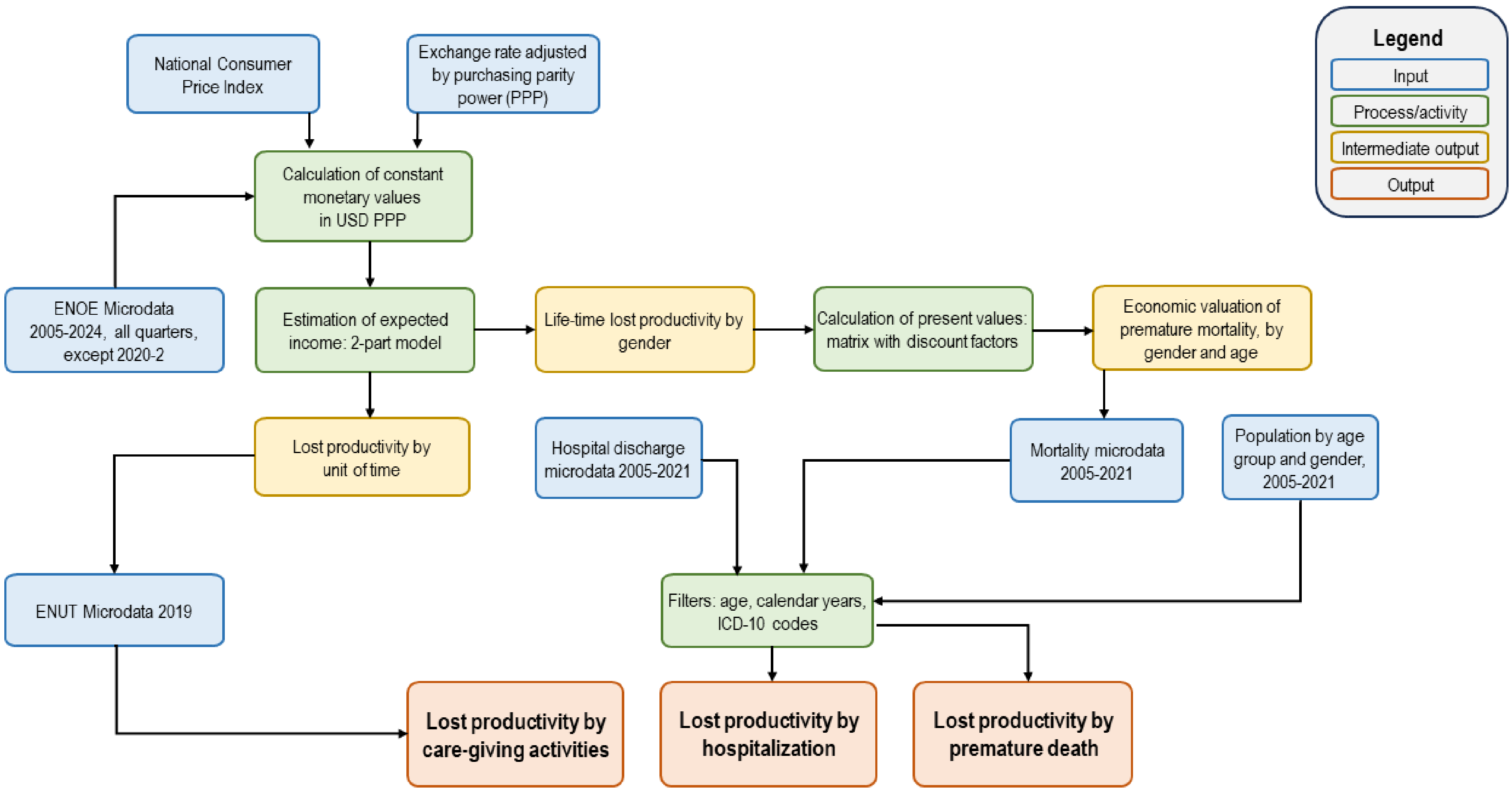

Note: The first step consisted in calculating the expected productivity (income) by sex/gender by unit of time using microdata from ENOE. We converted all monetary variables from ENOE to US$ PPP using the national consumer price index from INEGI and the exchange rate adjusted by purchasing parity power (PPP) from the OECD. The expected productivity is the result of multiplying the predicted probability of labor participation by the expected (predicted) income from labor after running Probit and linear regressions, respectively. This gave us the expected productivity by unit of time (hour). This expected productivity by age and

gender was the main input for estimating lost productivity by premature death and hospitalizations. Regarding premature mortality, for each gender and age, we calculated the present value of the sum of the expected annual contemporaneous and future income, given age and gender. On this basis, we were able to obtain an economic valuation of premature mortality by gender and age by inputting these expected present values into the mortality microdata. We did so by merging the values included in Appendix 2 -Table A, by gender, age and size of locality to the mortality microdata. We then calculated the total productivity loss by gender and set of diseases, using the ICD-10 codes detailed in the methods section. For hospitalizations, we used the hospital discharge information and the expected hourly productivity and proceeded in a similar fashion, using the same ICD-10 codes and the number of days at the hospital, assuming that one day equaled eight working hours and using the values in Appendix 3 - Table A2. Although it was not part of our study objectives, in the Discussion Section of this paper, we also estimated the productivity loss by time spent in care activities, using the National Survey on Use of Time 2019 (*ENUT*), as described in Appendix 4. Regarding the sensitivity analyses, as a result of an initial finding that figures for lost productivity were much higher among men, we needed to be certain that our results supported this conclusion. As discussed, the total amount of lost productivity was a function of two variables: burden of disease and expected productivity by unit of time. Accordingly, we calculated several scenarios of total productivity loss based on several assumptions: (a) that women and men enjoy the same average expected productivity by age, irrespective of gender/sex, and (b) that women have the same expected productivity as men. As discussed in the text, we found that in general, lost productivity was higher among men both because they faced a higher burden of disease, and because men have higher expected productivity (by probability of labor participation and income from labor given participation holds).

Appendix 2 - Table A1. Present value of forgone productivity by age, gender, and location size, 2021 Intl. US$. Mexico, adults 20-76y, 2005-2021

Age	Men			Women		
	Rural	Urban	All	Rural	Urban	All
20	123,844.0	156,404.1	138,519.1	61,312.3	75,963.1	100,857.0
21	123,423.9	156,059.1	138,132.8	60,810.6	75,435.0	100,348.0
22	122,760.7	155,418.4	137,468.6	60,177.6	74,713.2	99,620.4
23	121,884.9	154,523.1	136,567.9	59,412.8	73,837.8	98,703.8
24	120,892.4	153,399.2	135,499.8	58,579.2	72,850.1	97,662.9
25	119,749.4	152,099.9	134,270.8	57,670.7	71,755.7	96,501.4
26	118,526.5	150,638.0	132,927.2	56,727.7	70,589.5	95,264.2
27	117,211.8	149,048.5	131,468.0	55,743.7	69,358.4	93,946.0
28	115,801.8	147,326.7	129,899.4	54,712.3	68,076.6	92,556.8
29	114,309.9	145,521.6	128,255.7	53,644.6	66,749.8	91,113.8
30	112,706.8	143,606.8	126,503.8	52,526.0	65,351.8	89,592.9
31	111,090.3	141,611.9	124,712.7	51,419.0	63,963.6	88,065.3
32	109,359.8	139,510.6	122,812.7	50,233.3	62,518.0	86,451.6
33	107,548.9	137,290.1	120,814.7	49,013.1	61,037.1	84,775.3
34	105,655.3	135,003.3	118,753.7	47,795.8	59,533.7	83,066.9
35	103,651.4	132,571.6	116,571.4	46,536.2	57,973.7	81,281.3
36	101,590.1	130,070.4	114,320.9	45,261.7	56,389.2	79,456.6
37	99,442.9	127,509.0	111,996.8	43,960.6	54,777.0	77,583.5
38	97,225.7	124,877.0	109,601.6	42,615.6	53,140.6	75,655.3
39	94,972.1	122,169.4	107,148.7	41,265.9	51,499.6	73,700.0
40	97,620.0	125,638.4	110,171.6	42,414.9	52,890.0	75,761.7
41	95,388.5	122,944.4	107,735.5	41,116.1	51,326.8	73,848.8
42	93,016.4	120,154.4	105,170.1	39,782.7	49,733.4	71,859.5
43	90,539.6	117,253.6	102,495.9	38,383.9	48,113.5	69,790.7
44	88,047.0	114,346.8	99,810.0	37,029.0	46,486.7	67,734.7
45	85,470.9	111,344.7	97,040.3	35,677.0	44,853.1	65,647.0
46	82,871.2	108,279.8	94,220.9	34,322.4	43,220.7	63,532.0
47	80,248.5	105,139.2	91,357.0	32,964.0	41,577.9	61,389.9
48	77,511.9	101,894.6	88,376.5	31,574.0	39,947.2	59,193.2
49	74,787.8	98,647.8	85,402.9	30,220.9	38,318.1	57,013.3
50	72,020.8	95,366.9	82,387.7	28,887.6	36,705.7	54,830.6
51	69,261.7	92,071.0	79,355.3	27,596.3	35,121.4	52,661.0
52	66,447.4	88,646.4	76,237.9	26,291.3	33,520.0	50,442.9
53	63,590.1	85,175.8	73,079.8	24,992.0	31,917.2	48,212.1
54	60,757.3	81,700.1	69,939.2	23,730.9	30,363.2	46,018.8
55	57,921.7	78,208.0	66,793.3	22,507.5	28,846.4	43,852.3
56	55,134.9	74,662.5	63,652.9	21,293.2	27,319.9	41,684.6
57	52,347.2	71,115.0	60,502.1	20,096.7	25,825.5	39,528.1
58	49,559.3	67,529.2	57,336.7	18,922.0	24,356.5	37,379.8
59	46,741.6	63,932.0	54,149.6	17,777.4	22,907.4	35,243.6
60	43,902.1	60,294.4	50,941.5	16,621.1	21,468.4	33,098.0
61	41,162.3	56,683.8	47,794.6	15,513.2	20,045.4	30,993.4
62	38,412.3	53,056.2	44,631.9	14,422.2	18,641.3	28,895.3
63	35,604.1	49,480.1	41,460.7	13,303.3	17,241.4	26,782.8
64	32,887.1	45,870.6	38,331.5	12,218.7	15,878.7	24,713.6
65	30,220.6	42,311.9	35,266.2	11,156.1	14,563.6	22,697.3
66	27,621.3	38,754.9	32,244.6	10,158.5	13,282.4	20,737.0
67	25,002.0	35,211.9	29,214.9	9,187.2	12,008.2	18,786.0
68	22,409.7	31,676.8	26,206.2	8,203.8	10,742.2	16,834.5
69	19,820.2	28,134.3	23,212.8	7,257.3	9,497.7	14,906.8
70	17,224.4	24,553.5	20,194.8	6,301.0	8,267.1	12,968.7
71	14,675.2	20,955.5	17,212.6	5,362.2	7,061.5	11,056.7
72	12,172.0	17,414.8	14,289.0	4,433.5	5,831.3	9,160.7
73	9,682.7	13,911.3	11,373.9	3,491.6	4,632.1	7,275.0

74	7,217.8	10,442.4	8,499.0	2,602.8	3,466.8	5,440.4
75	4,791.8	6,939.3	5,646.4	1,732.3	2,296.9	3,617.5
76	2,411.1	3,456.8	2,822.2	865.3	1,146.7	1,809.2

Appendix 3 - Table A2. Expected hourly productivity, by age, gender, and location size, 2021 Intl. US$. Mexico, adults 20-76, 2005-2021

Age	Men		Women		All
	Rural	Urban	Rural	Urban	
20	2.091	2.547	1.184	1.419	1.787
21	2.208	2.691	1.243	1.509	1.890
22	2.305	2.810	1.300	1.576	1.975
23	2.351	2.912	1.323	1.620	2.024
24	2.412	2.984	1.348	1.658	2.069
25	2.435	3.047	1.352	1.678	2.090
26	2.463	3.089	1.358	1.693	2.112
27	2.491	3.132	1.367	1.700	2.128
28	2.511	3.148	1.370	1.704	2.134
29	2.545	3.176	1.379	1.719	2.151
30	2.527	3.187	1.357	1.693	2.132
31	2.560	3.211	1.380	1.701	2.152
32	2.575	3.239	1.379	1.697	2.159
33	2.589	3.239	1.359	1.686	2.150
34	2.616	3.278	1.362	1.692	2.163
35	2.615	3.276	1.350	1.681	2.156
36	2.627	3.268	1.345	1.671	2.152
37	2.630	3.265	1.347	1.658	2.152
38	2.614	3.263	1.329	1.636	2.136
39	2.635	3.272	1.307	1.624	2.130
40	2.609	3.268	1.300	1.593	2.117
41	2.647	3.276	1.298	1.584	2.126
42	2.663	3.290	1.311	1.574	2.136
43	2.634	3.249	1.267	1.552	2.098
44	2.638	3.253	1.245	1.531	2.083
45	2.611	3.239	1.226	1.506	2.065
46	2.583	3.231	1.208	1.486	2.047
47	2.601	3.236	1.203	1.455	2.042
48	2.553	3.188	1.163	1.430	2.000
49	2.534	3.156	1.133	1.397	1.969
50	2.488	3.113	1.091	1.358	1.929
51	2.474	3.128	1.079	1.343	1.920
52	2.453	3.100	1.056	1.319	1.893
53	2.397	3.050	1.017	1.270	1.840
54	2.356	3.005	0.979	1.228	1.794
55	2.288	2.979	0.955	1.210	1.761
56	2.246	2.927	0.928	1.170	1.723
57	2.204	2.892	0.899	1.135	1.686
58	2.177	2.843	0.866	1.102	1.647
59	2.145	2.809	0.854	1.075	1.620
60	2.051	2.740	0.812	1.045	1.566
61	2.015	2.694	0.787	1.014	1.531
62	2.003	2.613	0.785	0.991	1.507
63	1.914	2.576	0.750	0.951	1.453
64	1.847	2.495	0.723	0.906	1.394
65	1.773	2.441	0.674	0.869	1.336
66	1.744	2.379	0.645	0.846	1.301
67	1.690	2.322	0.637	0.822	1.272
68	1.649	2.272	0.603	0.792	1.230
69	1.613	2.237	0.594	0.766	1.206
70	1.550	2.192	0.570	0.735	1.164
71	1.488	2.108	0.551	0.729	1.126
72	1.443	2.036	0.544	0.695	1.093
73	1.393	1.965	0.502	0.660	1.038
74	1.336	1.930	0.480	0.644	1.004

75	1.277	1.866	0.465	0.616	0.969
76	1.256	1.800	0.451	0.597	0.942

Appendix 4. Estimation of time spent giving care to household members that require special assistance

To estimate of the time and lost productivity caused by spending time giving care, we used the 2019 National Survey on Use of Time (ENUT for its acronym in Spanish), collected by the National Institute of Statistics and Geography (INEGI). ENUT is nationally representative of population aged 12 and above with sample size of 71,404. We identified the variables associated with providing help, care and assistance to household members who need it (Feeding, helping to wear clothes, helping to carry the household members, helping to prepare, providing medicines, taking to seek health-care, providing therapy, and taking the household member to other activities) and calculated the weekly time spent in these activities. Using the expected hourly productivity by gender and age in Appendix 3, we also estimated the productivity loss as an opportunity cost by these activities assuming that individuals sacrificed their time when caring for other household members. We used the sample design to produce our estimates shown in Table A3. To provide annual figures, we assumed that 1 year = 52 weeks and that there is not seasonal component. Women spend 75% of the weekly time needed to perform these assistance activities compared to 25% of men. Women's opportunity cost (lost productivity) was estimated at 6.4 million Intl. US$ per week, whereas for men it was estimated at 4.4 million Intl. US$ per week. However, if we assumed equal productivity among women and men, the lost productivity of women would ascend to 13.4 million Intl. US$ per week and 699.2 million Intl. US$.

Appendix 4 - Table A3. Time spent in giving care. Mexico, adults aged 20-76y, by type of activity and sex, 2019

Activity	Men	Women	Women, asumming equal productivity
**Feeding**			
Prevalence, %	24.6	41.3	41.3
Weekly hours	0.70	0.92	0.92
Mean productivity loss (Intl. US$)	1.98	1.23	2.61
Total weekly hours	502,906.0	1,343,932.0	1,343,932.0
Total weekly productivity loss (Intl. US$)	1,431,266.0	1,796,355.0	3,816,236.0
Total annual hours	26,151,112.0	69,884,464.0	69,884,464.0
Total annual productivity loss (Intl. US$)	74,425,832.0	93,410,460.0	198,444,272.0
**Helping to wear clothes**			
Prevalence, %	15.3%	40.2%	40.2%
Weekly hours	0.46	0.62	0.62
Mean productivity loss (Intl. US$)	1.28	0.85	1.79
Total weekly hours	207,530.1	887,343.9	887,343.9
Total weekly productivity loss (Intl. US$)	573,017.5	1,209,743.0	2,542,554.0
Total annual hours	10,791,565.2	46,141,882.8	46,141,882.8
Total annual productivity loss (Intl. US$)	29,796,910.0	62,906,636.0	132,212,808.0
**Helping to carry**			
Prevalence, %	21.6	26.2	26.2
Weekly hours	0.33	0.56	0.56
Mean productivity loss (Intl. US$)	0.93	0.78	1.62
Total weekly hours	209,537.2	517,818.2	517,818.2
Total weekly productivity loss (Intl. US$)	591,841.1	726,936.2	1,501,840.0
Total annual hours	10,895,934.4	26,926,546.4	26,926,546.4
Total annual productivity loss (Intl. US$)	30,775,737.2	37,800,682.4	78,095,680.0
**Helping to prepare remedies**			
Prevalence, %	9.0	27.1	27.1
Weekly hours	0.36	0.32	0.32
Mean productivity loss (Intl. US$)	0.97	0.43	0.90
Total weekly hours	95,599.2	304,124.7	304,124.7
Total weekly productivity loss (Intl. US$)	256,770.8	411,505.0	865,740.2
Total annual hours	4,971,157.9	15,814,484.4	15,814,484.4
Total annual productivity loss (Intl. US$)	13,352,081.6	21,398,260.0	45,018,490.4
**Providing medicines**			
Prevalence, %	24.5	50.8	50.8
Weekly hours	0.25	0.29	0.29
Mean productivity loss (Intl. US$)	0.69	0.40	0.85
Total weekly hours	179,573.5	530,029.9	530,029.9
Total weekly productivity loss (Intl. US$)	497,951.5	720,679.7	1,519,728.0
Total annual hours	9,337,822.0	27,561,554.8	27,561,554.8
Total annual productivity loss (Intl. US$)	25,893,478.0	37,475,344.4	79,025,856.0
**Taking to health care**			
Prevalence, %	17.3	28.0	28.0
Weekly hours	0.53	0.72	0.72
Mean productivity loss (Intl. US$)	1.47	0.97	2.05
Total weekly hours	268,175.3	712,667.3	712,667.3
Total weekly productivity loss (Intl. US$)	744,570.0	960,599.5	2,030,006.0
Total annual hours	13,945,115.6	37,058,699.6	37,058,699.6
Total annual productivity loss (Intl. US$)	38,717,640.0	49,951,174.0	105,560,312.0
**Providing therapy**			
Prevalence, %	6.2	12.8	12.8
Weekly hours	0.06	0.10	0.10
Mean productivity loss (Intl. US$)	0.17	0.14	0.30
Total weekly hours	44,755.5	152,556.5	152,556.5
Total weekly productivity loss (Intl. US$)	120,359.9	208,307.0	436,532.2
Total annual hours	2,327,287.6	7,932,938.0	7,932,938.0

Total annual productivity loss (Intl. US$)	6,258,714.8	10,831,964.0	22,699,674.4
**Taking to activities**			
Prevalence, %	7.2	13.0	13.0
Weekly hours	0.28	0.53	0.53
Mean productivity loss (Intl. US$)	0.86	0.79	1.60
Total weekly hours	59,533.1	242,191.4	242,191.4
Total weekly productivity loss (Intl. US$)	180,410.9	334,064.9	732,831.4
Total annual hours	3,095,723.3	12,593,952.8	12,593,952.8
Total annual productivity loss (Intl. US$)	9,381,366.8	17,371,374.8	38,107,232.8
**Total**			
Total weekly hours	1,567,610.0	4,690,663.9	4,690,663.9
Total weekly productivity loss (Intl. US$)	4,396,187.7	6,368,190.3	13,445,467.8
Total annual hours	81,515,717.9	243,914,522.8	243,914,522.8
Total annual productivity loss (Intl. Million US$)	228.6	331.1	699.2
% of time	25.0	75.0	25.0
% of productivity loss	40.8	59.2	75.4

Note: Intl. Million US$=International Million United States Dollars.
